# Personals

**Published:** 1918-10

**Authors:** 


### PERSONAL

Announcement of removal, travel, and other matters of interest are requested. Please report errors in the listing of any physician in the State and other directories, that we may co-operate with the State Society in securing a correct list.

Dr. DeWitt H. Sherman of Buffalo visited in Shinnecock and Stockbridge, Mass., in August.

Dr. Edgar A. Forsyth of Buffalo motored through the Adirondacks late in August.

Dr. R. I. Palmer of Silver Creek has resigned from the local board of education of which he was president.

Dr. J. W. Fitz-Gerald of Buffalo spent August in Georgian Bay.

Dr. M. M. Cole of Newfane has accepted the position as assistant physician at the Soldiers' Home in Bath.

